# Genome variation in the *Batrachochytrium* pathogens of amphibians

**DOI:** 10.1371/journal.ppat.1012218

**Published:** 2024-05-23

**Authors:** Theresa Wacker, Nicolas Helmstetter, David J. Studholme, Rhys A. Farrer

**Affiliations:** 1 Biosciences, University of Exeter, Exeter, United Kingdom; 2 Medical Research Council Centre for Medical Mycology at the University of Exeter, Exeter, United Kingdom; University of Maryland, Baltimore, UNITED STATES

## Overview

**Fig 1:**
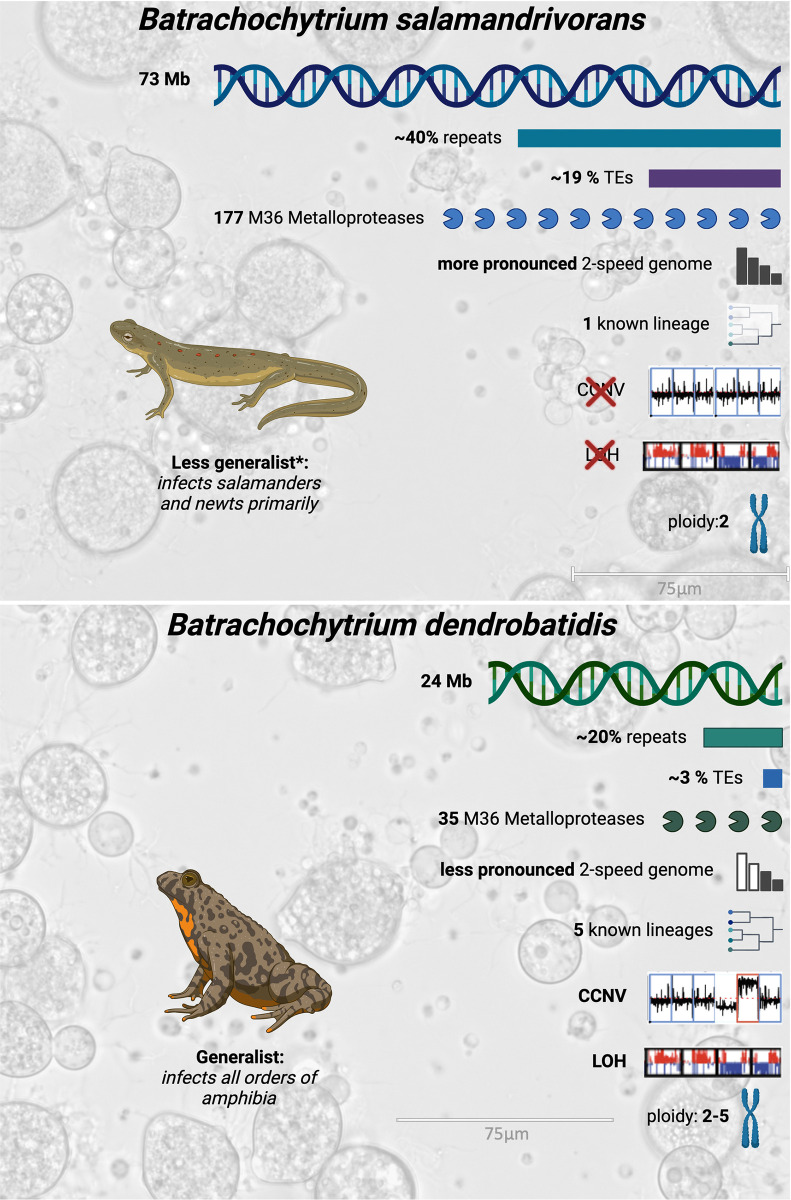
Overview of the major genomic differences between *Batrachochytrium salamandrivorans* (*Bsal*; top panel) and *Batrachochytrium dendrobatidis* (*Bd*; bottom panel). Amphibian illustrations and text below them highlight a key phenotypic difference between the pathogenic species. The background images for the species boxes are photos taken by an EVOS M5000 Imaging System at 40× magnification (scale bars shown) of *Bsal* (top panel) and *Bd* cells (bottom panel) grown in tGhL media. *In comparison to *Bd* based on the currently known host range. Figure created with BioRender.com.

Amphibians are the most endangered class of vertebrates, with over 40% threatened with extinction [1]. This biodiversity crisis is attributable to habitat loss, climate change and the chytridiomycosis panzootic. The causative agents, *Batrachochytrium dendrobatidis* (*Bd*) and *Batrachochytrium salamandrivorans* (*Bsal*) are the only chytrid fungi known to infect vertebrates [2,3], the majority being saprobes. Chytridiomycosis-associated 90 extinctions and 500 declines motivate a need to understand the evolution, pathophysiology, and pathogenicity of these 2 pathogens [4]. While both batrachochytrids can infect amphibia, they differ in host-range, distribution, and pathophysiology. *Bd* is a generalist that infects all orders of amphibia, is distributed worldwide, and causes hyperplasia and hyperkeratosis in the susceptible host. *Bd* has genetically diverse populations, comprising 5 known lineages to date: *Bd*GPL, *Bd*ASIA-1, *Bd*ASIA-2, *Bd*ASIA-3, and *Bd*CAPE [5,6]. Conversely, *Bsal* causes disease in, primarily, the Urodela order of amphibia (salamanders and newts), is currently only found in Europe and Asia, and causes multifocal superficial erosions and deep ulcerations of the skin of hosts [7]. Experimentally, *Bsal* has also been shown to be able to infect some anurans and cause disease in at least 2 frog species [8]. To date, only 1 lineage of *Bsal* has been identified. The genomic mechanisms underlying those differences are only partially understood because, to date, neither batrachochytrids are genetically tractable, although promising recent work has shown transient (persisting up to 3 generations) genetic transformation of *Bd* [9]. Here, we review the current state of knowledge based on genomics of batrachochytrids (Fig 1).

## 1) What are the main features of the batrachochytrid genomes?

*Bd* was identified as the cause of amphibian chytridiomycosis in 1999 [10]. The first genome sequence came from *Bd* isolate JEL423 belonging to the Global Panzootic Lineage (*Bd*GPL) in 2006, using Sanger sequencing [11]. The resulting genome assembly of 69 supercontigs had a total length of 23.7 Mb; annotation aided by RNA-seq identified 8,630 protein coding genes across 9,893 transcripts [11]. Three further *Bd* genome assemblies have been deposited in GenBank, including *Bd*GPL isolates JAM81 and RTP6 and *Bd*BRAZIL CLFT071, all with similar lengths (22.2 Mb to 23.8 Mb). The species includes diploid and triploid strains with common copy-number variations (CNV), including chromosomal CNVs (CCNV) [12]. Recently, pan-genomic analysis was performed, revealing lineage-specific genes including pathogenicity genes [13].

*Bsal* was discovered more recently (in 2013) as the infectious agent of mass mortality in Dutch fire salamanders, reducing their population to 4% of their original size within 3 years [2]. Isolate AMFP13 from that outbreak was sequenced, initially with short reads and later refined using nanopore sequencing [14]. The resulting genome assembly was substantially longer than those of *Bd*, comprising 73.3 Mb in 165 supercontigs and encoding 10,867 protein-coding genes. Eight additional genome assemblies from different outbreak sites, infected captive animals, and multiple samples at different time points from the same populations provided evidence of potentially drastically variable genome lengths (as small as 27.6 Mb, albeit in thousands of contigs) [15]. In common with some strains of *Bd*, *Bsal* is diploid, although no triploid isolates have been identified to date and it has fewer CNVs [14,15].

## 2) What are the population structures of the batrachochytrids?

*Bd*’s 5 known lineages, including *Bd*GPL, *Bd*ASIA-1, *Bd*ASIA-2, *Bd*ASIA-3, and *Bd*CAPE [5,6], are largely genetically isolated and genetically diverse (e.g., high nucleotide diversity, π) [6]. A genotype named *Bd*CH was initially thought to represent a sixth lineage but was later found to group with *Bd*ASIA-1 and is referred to as ASIA-1-like [6]. Of the 5 lineages, *Bd*GPL is hypervirulent and globally distributed and the main driver of the chytridiomycosis panzootic [6]. *Bd*Asia-1 is the only lineage that shows mutation-drift equilibrium and a Tajima’s D of 0, indicative of endemism; all other lineages diverge from equilibrium values of Tajima’s D, indicative of outbreak dynamics [6]. Despite having no known mating-type alleles and lacking many components in the “meiotic toolbox” for meiotic recombination, *Bd* recombinants have been identified that may be the product of ancestral recombination or “parasexual” mitotic recombination [16]. Parasexual mitotic recombination could be responsible for the existing hybrids of *Bd*, but no mating or hybridization has been observed in the wild or laboratory settings so far [17].

No clearly demarcated lineages have been identified in *Bsal*, notwithstanding genomic differences among isolates from different infection sites and animals [15]. Indeed, *Bsal* features high genomic diversity, with more between- than within-outbreak divergence [15], suggesting multiple introductions of *Bsal* to Europe, similar to the situation for *Bd* [5,15]. There is also no evidence of sexual reproduction to date in *Bsal*.

## 3) What are the main differences between the batrachochytrid genomes?

Generalist pathogens often have larger genomes than those of relatives with a more restricted host range (e.g., *Fusarium* and *Metarhizium* species). Batrachochytrids show striking differences in genome lengths that oppose this trend: The 73.3-Mb genome assembly for the more host-selective *Bsal* (at least, compared with *Bd*) is 3 times longer than that for the generalist *Bd* (23.7 Mb). The evolutionary cause of this difference in genomic length is currently unclear but could reflect population dynamics or perhaps reflect that *Bsal* is also more of generalist than currently experimentally ascertained [8]. In either case, it is unclear whether *Bsal* has undergone a genome expansion, or if *Bd* has undergone a contraction. Possible evolutionary explanations for changes in genome size include founder effects in *Bsal* (i.e., a reduced effective population size) related to its recent introduction in Europe, perhaps coinciding with a reduction or loss of effective control of transposable elements (TE) proliferation.

While the genome of *Bd* is shorter than that of *Bsal*, *Bd* has a greater degree of variability in ploidy and aneuploidy [12]. Little is currently known about the mechanisms regulating ploidy in the chytrids. However, if this genomic variation is a source of phenotypic plasticity in *Bd*, it may underpin its ability to infect a wide range of hosts [12]. Some genomes of *Bd* harbour an endogenous DNA virus (BdDV-1) that reduce *in vitro* growth but increase virulence [18], while no mycoviruses have been discovered in *Bsal* to date.

The batrachochytrids genomes differ in the profiles of repetitive DNA with *Bsal* genome assemblies consisting of between 21% and 41% repeats compared with 18% in *Bd*. Approximately half of the repeats in *Bsal* are TEs [14], amounting to 10-fold more than in *Bd* [14,15]. The reference genome for *Bsal* (AMFP13) is the most repeat- and transposon-rich genome among the Chytridiomycota sequenced to date, with a higher repeat content than the average of 5% to 35% observed in most fungi [19]. Long terminal repeat (LTR) and long interspersed nuclear element (LINE) retrotransposons are the most abundant repeat superfamilies in *Bsal*, with many retaining domains required for activity. Conversely, LTR and LINE elements are almost completely absent from the *Bd* genome and none are fully functional or autonomous.

## 4) What are the major virulence factors in the batrachochytrids and how do they differ between the 2 species?

Both *Bd* and *Bsal* encode several expanded gene families, most notably the aspartyl proteases and the M36 metalloproteases. The M36 family expansion is far more pronounced in *Bsal* compared with *Bd* (*n* = 177 versus 35) [11,14]. Genes encoding M36 metalloproteases are up-regulated during infection *in vivo* in both *Bd* and *Bsal*, and these proteases are thought to play a role during host invasion via skin and extracellular matrix destruction [11]. Regions of *Bd*’s genome associated with CCNV are enriched for genes encoding serine and aspartic proteases, protein families that have been previously implicated in host invasion. These gene families are expanded in *Bd* and under positive selection [12,20].

An enigmatic expanded family of proteins encoded by the batrachochytrids and saprobic relatives [11] have sequence similarity to the crinkling and necrosis (CRN) genes more extensively studied among several species in the Oomycetes. That *Bd* encodes as many as 162 CRN-like genes, compared with only 10 in *Bsal*, suggests some deviation in function and/or evolutionary selective pressures. Additionally, while CRN-like genes show an increased expression in *Bd* zoospore-infected host tissue, the expression was decreased in *Bsal*, indicating their importance in early infection in *Bd*, but not *Bsal* [11]. In *Bd*, CRN-like genes are among the core gene set up-regulated in both highly and less or nonsusceptible hosts during infection, together with peptidases, carboxypeptidases, and metalloproteases with signal peptides [21].

Another groups of proteins thought to be involved in host–pathogen interactions and adhesion are the carbohydrate binding proteins (CBMs), in particular the lectin-like class of CBM18s [11]. The CBM18 gene family is expanded in the batrachochytrids, especially in *Bd*, and its members show length and sequence divergence between *Bd* and *Bsal* [11]. In *Bd*, CBM18s are thought to play a role in dampening chitin-based host recognition, but as they are pronouncedly truncated in *Bsal* and appear to be absent in some isolates, this might not be the case in *Bsal* [11,15,22]. Conversely, in *Bsal*, galactose-binding ricin B-like lectin CBMs play an important role in early pathogenesis by mediating chemotaxis, adhesion, and virulence [23]. This does not appear to be the case for *Bd*. Notably, cell adhesion and cell projection genes are up-regulated in susceptible hosts of *Bd*, but not in less or nonsusceptible hosts [21].

## 5) What is a “two-speed genome” and do the batrachochytrids have one?

While TEs can have negative fitness consequences for the host, they are also a source of genomic innovation and rapid adaptation [24]. An evolutionary strategy for balancing the conflicting drivers for maintaining and eliminating TEs is having a “two-speed” genome, portioned into subgenomic compartments that are rich in repeats and rapidly evolving, and into other subgenomic compartments that are repeat-sparse and gene-dense and more slowly evolving. Both the batrachochytrids display the hallmarks of a two-speed genome, which are more pronounced in *Bsal* [14]. Dynamic regions of the *Bsal* genome show stronger enrichment of genes for M36 metalloproteases, secreted proteins, and proteins up-regulated during infection compared to *Bd* [14]. In contrast, core, conserved genes are found in the stable, TE- and repeat-sparse compartments in both batrachochytrids [14]. Intriguingly, several LINE families are significantly enriched upstream of the largest expansion of M36 genes (family 6), suggesting these LINEs were responsible for that expansion [14]. Meanwhile, genes with signatures of positive or relaxed selection in each of the *Bd* lineages (*dN/dS* (ϖ) > 1) have significantly longer flanking intergenic regions. Together, this reveals the importance of repetitive elements to the genome evolution and predicted pathogenicity genes in the batrachochytrids.

## 6) What are the future directions in batrachochytrid genome research?

The batrachochytrids have two-speed genomes, where repeat-rich regions of the genome are enriched in putative virulence genes, genes under positive selection, and genes up-regulated *in vivo*. However, large genomic regions under either evolutionary speed are not found in the batrachochytrids, whereas accessory chromosomes, compartments found in telomeres and sub-telomeres and lineage-specific regions have previously been described in some other species with a two-speed genome. Conversely, smaller-scale or even 3D (chromatin structure) compartmentalization has been poorly resolved and may yield new insight into genome organisation of the batrachochytrids and other species with two-speed genomes. Currently, only 1 *Bsal* assembly (GCA_002006685.2) features resolved telomeres on some but not all contigs [14]. The same is true for *Bd* [25]. Thus, to draw reliable conclusions on both the genome architecture and evolution of *Bsal* and *Bd*, improved reference genome assemblies are paramount.

The genetic interactions occurring between batrachochytrids and other microorganisms are poorly understood. The recent discovery and characterisation of the *Bd* mycovirus BdDV-1 provides a tantalising insight and opportunity to further explore the importance of these interactions. Additional sampling and genomic comparisons to other non-batrachochytrids is also necessary for understanding batrachochytrid evolution.

Gene regulation in the batrachochytrids are understudied, including lineage-specific differences during infection. Characterisation of transcription factors or other regulatory elements in the batrachochytrids that govern putative virulence genes such as the M36 metalloproteases, CRNs, or CBM18s has not been demonstrated. Indeed, no experimental evidence for the function of those genes has been reported, which is essential for determining their evolution and the importance of individual genes in those large gene families. Each of these research questions could provide important advances that lead to new approaches to counter chytridiomycosis.

Elucidating the full host-range of *Bsal* is important to facilitate a better assessment of potential threats for amphibian populations, support conservation efforts, and draw further conclusions on epidemiology and population structure of *Bsal*. Sampling efforts to assess the spread and host-range of *Bsal* might also yield further evidence of the mode of reproduction of *Bsal*. Additionally, with the suspected batrachochytrid pandemics originating in Asia for both *Bd* [6] and *Bsal* [26], further screening and sampling efforts, especially focused in Asia, may reveal novel lineages of *Bsal* and further additional lineages of *Bd*. The identification of these new lineages, especially those that are basal and harbour phenotypic differences, can provide opportunities to understand the molecular mechanisms governing those traits through comparative genomic and gene modifying approaches.
